# Beyond pulmonary vein isolation for persistent atrial fibrillation: sequential high-resolution mapping to guide ablation

**DOI:** 10.1007/s10840-021-01115-7

**Published:** 2022-01-08

**Authors:** Katarzyna Malaczynska-Rajpold, Julian Jarman, Rui Shi, Piers Wright, Tom Wong, Vias Markides

**Affiliations:** 1 Royal Brompton & Harefield Hospitals, Guy’s & St Thomas, NHS Foundation Trust, London, UK; 2 National Heart & Lung Institute, Imperial College London, London, UK

**Keywords:** Persistent atrial fibrillation, Sequential activation mapping, Individualized mapping, Focal activity, Rotational activity

## Abstract

**Purpose:**

We aimed to evaluate whether outcomes with ablation in persistent (PsAF) and long-standing persistent (LsPsAF) AF can be improved beyond what can be achieved with pulmonary vein isolation (PVI) alone, using individualized mapping to guide ablation.

**Methods:**

We studied 20 pts (15 M, 68 ± 11y) with PsAF (14) or LsPsAF (6) referred for first-time AF ablation. Following antral PVI, individualized mapping (IM) was performed using a high-density mapping catheter stably and fully deployed for 30 s at each of 23 ± 9 sites per patient. Activation data were reviewed, and an ablation strategy designed to intersect areas of focal and rotational activity. Mean follow-up was 429 ± 131 days. The study population was compared to a matched contemporary control cohort (CC) of 20 consecutive patients undergoing conventional ablation.

**Results:**

Despite the IM group having a higher median comorbidities score, 3.5 vs. 2.5 in the CC group, indicating potentially more complex patients and more advanced substrate, cumulative freedom from AF after a single procedure was achieved in 94% of patients in the IM group vs. 75% in the CC group at 1 year and remained the same in both groups at the conclusion of the study (*p* = 0.02). There was a similar trend in atrial arrhythmia-free survival between both groups (84% vs. 67% at 1 year) that did not reach statistical significance. The procedure duration was longer in the IM group by a median of 31.5 min (*p* = 0.004).

**Conclusions:**

Individualized mapping to guide AF ablation appears to achieve significantly greater AF-free survival compared to conventional PVI when applied as a primary ablation treatment. The results of this pilot study need to be confirmed in a larger, randomized trial.

**Supplementary Information:**

The online version contains supplementary material available at 10.1007/s10840-021-01115-7.

## Introduction

Pulmonary vein isolation (PVI) has become the cornerstone of atrial fibrillation (AF) ablation procedures. With technological advancements mainly improving the reliability of acute ablation lesion formation, the success rate in patients with paroxysmal AF can reach up to 72% with a single procedure and up to 90% with multiple procedures [1]. However, the results are far from ideal in non-paroxysmal AF, with single procedural success rates often below 50% at 1 year and at best reaching 70–80% after multiple procedures [2]. Improving outcomes with ablation of non-paroxysmal AF beyond PVI has proved challenging, with complex fractionated atrial electrogram (CFAE) ablation and empiric linear ablation not improving outcomes in a large randomized study [3]. Individual patient mapping to guide ablation is highly attractive, aiming to address the drivers of AF in an individual beyond PVI, but poses challenges in catheter design, including a balance between global mapping and resolution, electrode contact, signal processing, and interpretation.

In this study, we assessed the potential for individualised, live, intra-procedural mapping to guide ablation beyond PVI, to help improve outcomes in patients with persistent (PsAF) and long-standing persistent AF (LsPsAF).

## Methods

A total of 21 patients with PsAF or LsPsAF presenting for AF ablation were recruited to this pilot study between April 2018 and December 2019. Written informed consent was obtained from all patients, and the study was approved by the institutional research committee. Exclusion criteria included age below 18 years and not presenting in AF at the time of the procedure. One patient was excluded because the AF was terminated during PVI. Baseline patient characteristics were compared to those of a matched contemporary conventionally treated control cohort (CC) of 20 consecutive patients undergoing ablation for non-paroxysmal AF ablation with contact force sensing catheters by the same operators at our centre between January and December 2017. Only de novo procedures were included. The previously validated FLAME score[4] was used to compare the complexity of patients and ablation substrate. It is an easily calculated method predicting single and multiple procedural outcomes for non-paroxysmal AF ablations based on the left atrial size and comorbidities. In patients with a high score, even multiple procedures are usually ineffective.

### Electrophysiologic procedure

All procedures were performed under general anaesthesia, using the CARTO 3 mapping system (Biosense Webster Inc., Irvine, CA) with the CARTOFINDER module. Mapping was performed using a 4–4-4 mm 20-pole PentaRay™ NAV catheter. A ThermoCool® SmartTouch® catheter was used for ablation (Biosense Webster Inc.), and a decapolar Dynamic XT™ catheter (Boston Scientific, MA) was placed in the CS.

AF cycle length (AFCL) was measured over 10 s in the distal CS, where there was a strong correlation with post-PVI left atrial appendage (LAA) cycle length (r = 0.76, *p* < 0.001). Hence, comparisons were made of AFCL in the distal CS at baseline, post-PVI and pre-cardioversion[5].

### Mapping of AF

Following PVI, multiple 30 s recordings of left atrial unipolar electrograms were sequentially acquired using a stably deployed PentaRay Nav catheter with well-apposed, fully open splines applied to the left atrial endocardium. By appropriate flection of the PentaRay catheter and differential alignment of the sheath, including where appropriate catheter inversion, we ensured stable deployment with the splines fully open and excellent contact with the left atrial endocardium, as corroborated by the shape of the PentaRay and electrograms. The CARTOFINDER system performs QRS subtraction on the electrograms (EGMs) to remove ventricular far-field ventricular signals, leaving only atrial signals to be annotated. For each unipole, a bipolar electrogram window is created from the two nearest electrodes and unipolar signal annotation is performed within this window using wavelet analysis. The local activation (LAT) time is displayed in a dynamic fashion relative to the current time within the 30-s recording. CARTOFINDER then creates “4D” activation maps during a 250 ms window referencing each electrogram in relation to all the other electrograms from all the electrograms of the PentaRay NAV catheter. This window then moves through the 30-s recording to display a changing activation map over time to depict wavefront propagation[6]. Unipolar electrograms and annotations from each PentaRay NAV electrode are displayed on the system alongside the 4D maps to allow the electrophysiologist to oversee the process (Supplementary videos 1 and 2).

### Identification of areas of interest

A mean of 23 ± 9 recordings were taken per patient. Recordings were often overlapping to ensure consistency and coverage.

#### Visual analysis of the recordings

After completion of the acquisition of all post-PVI mapping recordings, 4D activation maps and electrograms were visually reviewed to identify repetitive atrial activation patterns (RAAP). Areas demonstrating repetitive focal activation patterns (FAP) or rotational activity patterns (RAP) were marked and included as ablation targets. They were later compared with areas of RAAP identified by the automated algorithm in the CARTOFINDER module described below.

#### Automated algorithm to identify RAAPs

**Fig. 1:**
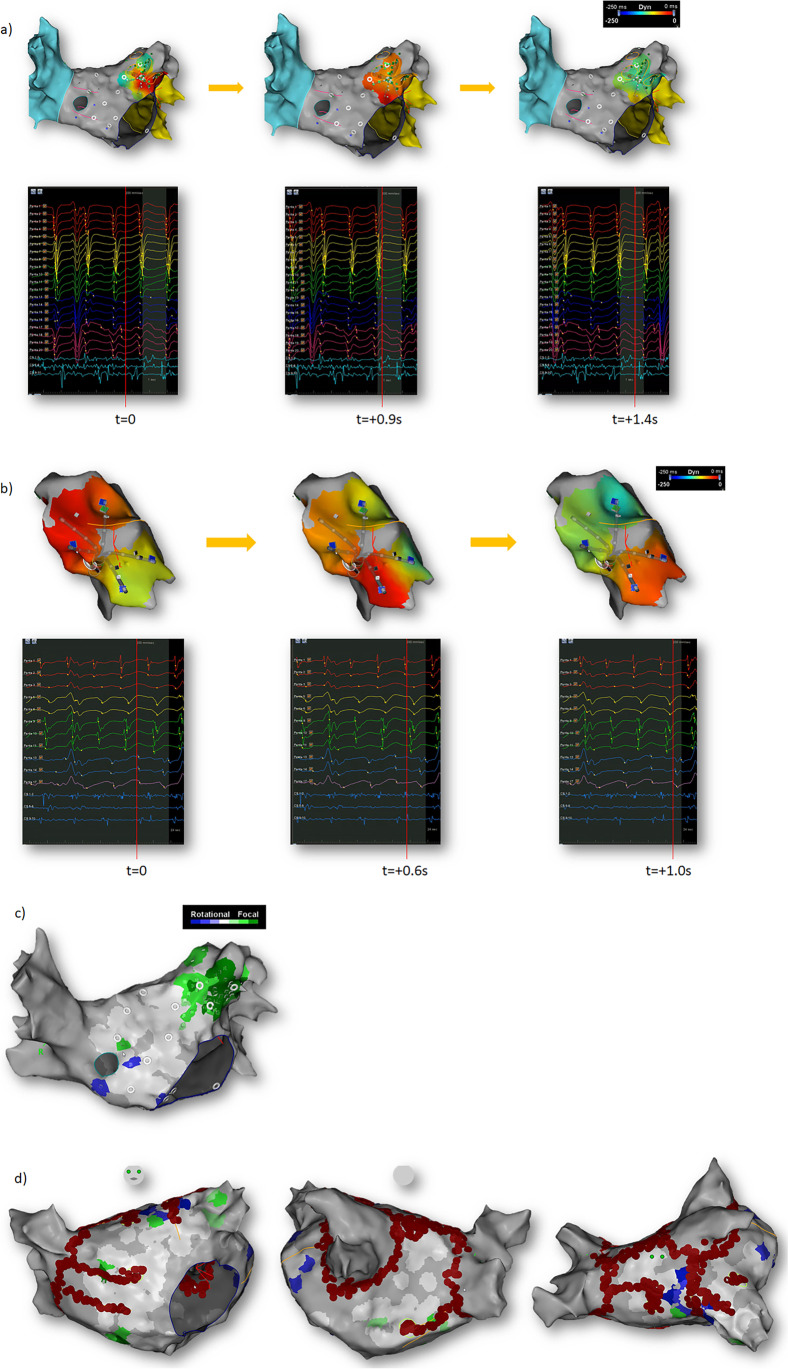
Automated annotation of focal (**a**) and rotational (**b**) activity by the CARTOFINDER module. (**c**) Green and blue color mark FAP and RAP, respectively. Color intensity represents the number of repetitions of the pattern during 30-s recording. (**d**) Example of a lesion set in AP, PA and superior views, respectively

Repetitive FAPs are defined as having radial activation over two or more cycles. The automated algorithm takes any earliest unipolar electrogram with a QS morphology within a 10-mm radius and 50 ms window prior to its annotation. Given the regional acquisition of data, the algorithm rejects points if they originate from a distal electrode. If the pattern occurs for two or more consecutive beats, the site of electrode recording is marked in green (Fig. 1c), with more repetitions within 30 s resulting in darker shades, and the time intervals during which they occur highlighted on the electrogram map (Fig. 1a, Supplementary video 1).

Repetitive rotational activation is defined as having 2 or more rotations of 360° within the recording. To identify such patterns, CARTOFINDER utilizes the PentaRay catheter design to assess ring formation by grouping electrodes from each spline located at a similar position along the spline. For each group of channels, the algorithm seeks “head meets tail” formation with certain conditions ensuring spatial and temporal continuity before determining whether the propagating wavefront and corresponding electrograms meet the criteria for rotational propagation. Temporal continuity requires the activation duration of the unipolar electrode group to be more than 50% of the acquisition CL. Spatial continuity is achieved by allowing no more than 20-mm distance between electrode locations having consecutive activation timing. Rotational conduction occurring for 2 or more consecutive beats is depicted with blue electrode colouring (Fig. 1c), with increasing rotational events during a 30 s recording shown as darker shades (Fig. 1b, Supplementary video 2). The algorithm has been validated in two independent studies[7, 8].

### Ablation strategy

An ablation strategy was planned using design lines outlining areas of focal activity and intersecting areas of rotational activity, and delivered, joining to anatomical/electrical barriers on at least one side of the line for all targeted rotational patterns, and for focal activity, in addition to targeting the focus and immediate surrounding area, joining to barriers where the focus was nearby to avoid the creation of narrow isthmi. Limited ablation of focal activity was performed within the LAA when necessary, often concentrating on the mouth of the LAA (Fig. 1d).

### Ablation technique

Ablation was performed using the ThermoCool® SmartTouch® catheter with a target contact force of 10 g (5–20 g) at 30 W for 30 s for the first 12 patients and 50 W/10–12 s thereafter[9].

### Procedure endpoints

The procedure was ended once the pre-planned lesions were delivered, or if AF terminated to sinus rhythm (SR). If AF organized to AT, this was mapped and ablated, but if AF continued, AFCL was measured, and DC cardioversion was performed. Finally, PVI and integrity of any linear ablation joining to two electroanatomical barriers were confirmed during SR and pacing.

### Control cohort–ablation protocols

The ablation technique used in the control cohort of 20 patients involved 30–40 W (25-35 W on the posterior wall) RF applications for 30 s, with a target contact force of 10 g (5–20 g). Two patients had conventionally performed antral PVI alone, while in the remaining patients, a variety of additional strategies were used, including roof (85%), posterior line (50%), mitral isthmus lines (20%), CFAE ablation (20%), endocardial CS roof ablation (25%) and epicardial ablation within the CS (20%).

### Follow-up

Follow-up was a mean of 429 ± 131 days in the study group and 482 ± 163 days in the control group (*p* = NS) including a 90-day blanking period. Patients had at least two follow-up visits: at 3 months and 1 year after the procedure. The long-term outcome of the ablation procedure in both groups was assessed based on a 7-day ECG Holter monitor performed around 1 year from the procedure, 12-lead ECG at the time of clinic follow-up appointments, and patient-reported symptoms. Patients were considered AF-free in the absence of symptoms suggestive of AF including palpitations (> 30 s) and absence of AF on ECGs and 7-day recordings beyond the 90-day blanking period.

### Statistical analysis

Statistical analyses were performed using STATISTICA 13.3 (StatSoft Power Solutions Inc., Poland). Continuous variables are given as mean ± standard deviation (SD) for normally distributed variables, or median (interquartile range) for non-normally distributed variables. The Student *t* test, or its non-parametric equivalent the Mann–Whitney U test where appropriate, was used for comparison of continuous variables. Fisher’s exact test was used for comparison of nominal variables. Logistic regression was used to assess probability to predict outcomes. Kaplan–Meier curves were used to show event-free survival, and a Cox’s F test was used to compare event-free survival rates between groups. Differences with a two-tailed *p* value below 0.05 were considered significant.

## Results

Baseline clinical characteristics are shown in Table 1. Overall, the two groups were well matched for each of the most relevant variables and comorbidities, including age, gender, body mass index, left atrial size and left ventricular function.

**Table 1:** Characteristics of patients presenting for AF ablation procedure in the IM vs. CC group

	Individualized mapping group (*n* = 20)	Control cohort (*n* = 20)	p
Age [yrs]	67.7 ± 10.9	63.5 ± 9.6	0.19
Male	15 (75)	16 (80)	1.0
BMI kg/m^2^	30.5 (28.0–34.0)	30.0 (26.5–33.5)	0.98
Co-morbidities			
Sleep apnoea	3 (15)	2 (10)	1.0
Hypertension	13 (65)	10 (50)	0.52
Diabetes	1 (5)	3 (15)	0.60
Obesity	11 (55)	11 (55)	1.0
CAD	3 (15)	3 (15)	1.0
Valvular disease	3 (15)	1 (5)	0.43
FLAME score	3.5 (3.0–4.0)	2.0 (1.0–4.0)	**0.026**
LV EF [%]	51 (35–60)	56 (46–60)	0.57
LA indexed volume [ml/m^2^]	42.8 ± 12.7	48.1 ± 15.8	0.26
PsAF/LsPsAF (> 1 yr)	14 (70)/6 (30)	12 (60)/8 (40)	0.74
Total AF duration [months]	28 (11–84)	34 (23–56)	0.38
Persistent AF duration [months]	9.5 (5.5–13.5)	13.5 (6.0–24.0)	0.27
Follow-up duration [days]	429 ± 131	482 ± 163	0.29
Antiarrhythmic drugs at follow-up	3 (15)	3 (15)	1.0

Values represent n (%), mean ± SD or median (IQR). *BMI* body mass index*, CAD* coronary artery disease, *FLAME* score predicting outcomes of non-paroxysmal AF ablation[4], *LV EF* left ventricular ejection fraction, *LA* left atrium, *PsAF* persistent AF (1 week to 1 year), *LsPsAF* long-standing persistent AF (above 1 year)

However, the FLAME score was higher in the IM group (median of 3.5 vs. 2.5, *p* < 0.026) suggesting that worse outcomes could have been expected in this group despite conventional matching of relevant variables. Unsurprisingly, the procedure duration was slightly longer in the IM group by a median of 31.5 min [IM 164.5 (154.–198.5) min vs. CC 123 (99–157) min, *p* = 0.004), while the ablation technique (30 W, longer duration vs. 50 W short-duration lesions) did not influence the long-term outcomes in the IM group significantly (*p* – NS).

Based on a mean of 23 ± 9 recordings per patient, the automated algorithm identified RAAPs in all patients. However, while focal activity was found in all, rotational activity was only demonstrated in 42%. A mean of 6.6 ± 3.6 (range 1–13) areas of focal and 0.8 ± 0.8 (range 0–2) of rotational activity per patient were demonstrated. The intensity of 42.3 ± 64.9 repetitions per activation source per patient was identified in FAPs and 7.1 ± 2.3 in RAPs. The most common locations for both FAPs and RAPs were the left atrial appendage and its base. Frequent RAAPs were also observed on the anterior wall towards the right-sided WACA line, on the roof and low posterior wall. Areas of RAAP were targeted with ablation lines or spokes anchored to an electrical barrier. A comparison of lesion sets in both groups is presented in Table S1 (supplementary material).

**Fig. 2:**
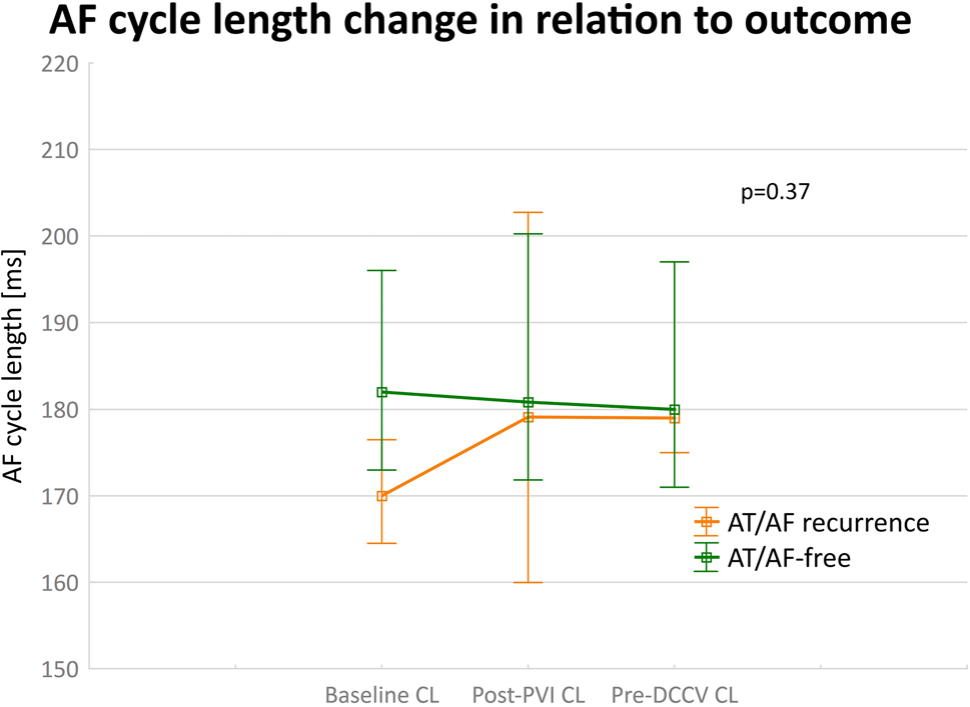
Changes in the atrial fibrillation cycle length (AFCL) in relation to long-term procedure outcome in the individualized mapping group (*n* = 20)

AFCL analysis in the IM group showed no significant difference in AFCL (pre-PVI) between those patients who remained arrhythmia-free and those with recurrence, median 182 (173–196) vs. 170 (164.5–176.5) ms respectively, *p* = 0.1) (Fig. 2). A univariate model did not show that the AFCL at baseline, post-PVI, or pre-DCCV was related to long-term freedom from AT/AF (*p* = 0.37).

**Fig. 3:**
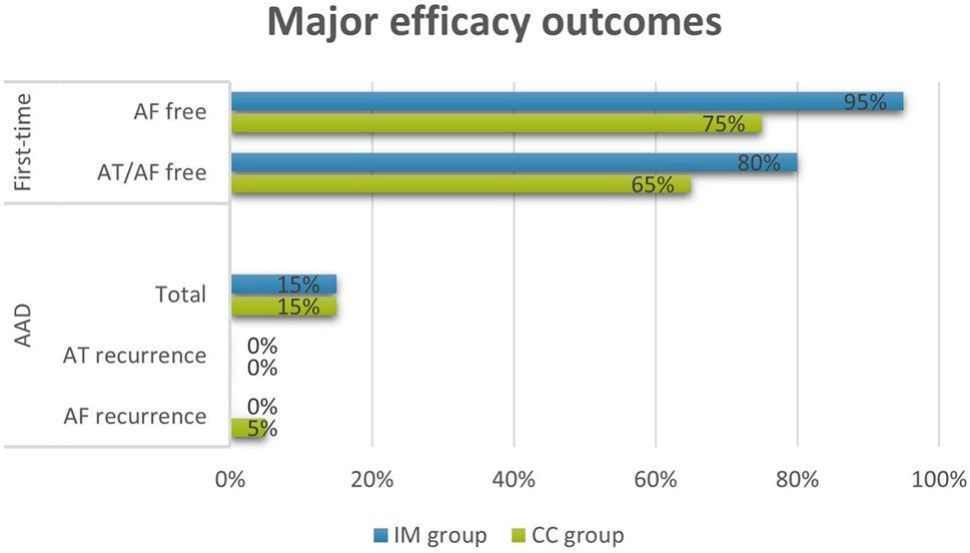
Major efficacy outcome at the end of the study and antiarrhythmic drugs (AAD) use on follow-up in the study group (*n* = 20) vs. control cohort (*n* = 20) show a trend towards superiority of individualized mapping in the first-time procedures

Cumulative freedom from AF at 1 year was achieved in 94% in the IM group vs. 75% in CC (*p* = 0.02), and freedom from AF/AT was achieved in 84% in the IM group vs. 67% in CC (*p* = 0.1). In each group, 3 patients (15%) remained on antiarrhythmic medication at the end of follow-up (Fig. 3).

**Fig. 4:**
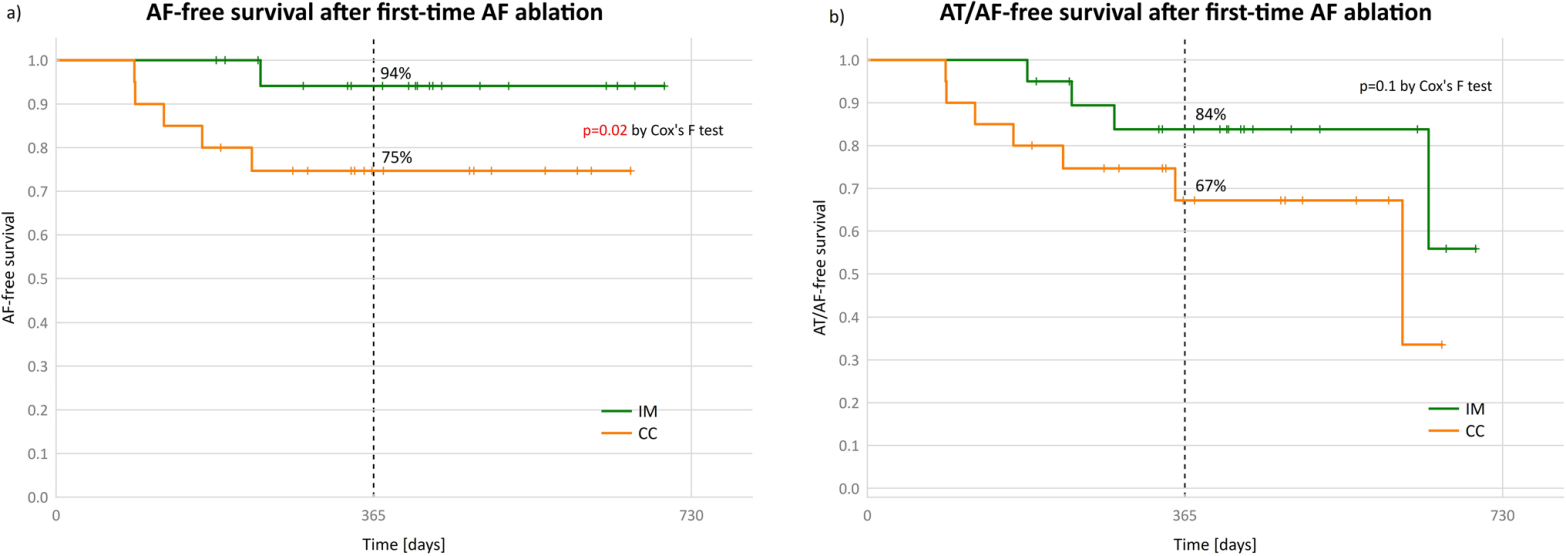
Cumulative AF-free (**a**) and AT/AF-free (**b**) survival after first-time AF ablation with or without antiarrhythmic drugs in the individualized mapping group compared to the contemporary control cohort

Comparisons of outcomes between IM and CC groups including the use of anti-arrhythmic drugs at the end of follow-up are shown in Table 2 and Fig. 3, and cumulative results are shown in Fig. 4, with cumulative AF-free survival of IM patients undergoing first-time ablation being significantly better than CC.

**Table 2:** Major efficacy outcomes at the end of follow-up

	Individualized mapping group ***n* = 20**	Control cohort ***n* = 20**	p
Freedom from AF	19 (95)	15 (75)	0.18
Freedom from AF/AT	16 (80)	13 (65)	0.48

Values represent n (%)

No procedural complications were noted in the IM group and one pseudoaneurysm was the only procedural complication in the CC group.

## Discussion

### Main findings

A primary application of IM-guided RF ablation for first-time non-paroxysmal AF resulted in 94% cumulative AF-free survival at 1 year. Automated post-PVI maps identified focal drivers in all patients, while rotational activity was seen in less than half. There was no significant difference in AFCL in relation to outcome, but a trend toward shorter baseline AFCL (pre-PVI) was observed in patients with worse long-term outcomes. Overall AFCL changes during the procedure did not predict long-term outcomes.

### Earlier studies

There have been numerous efforts over the past two decades to improve outcomes in non-paroxysmal AF. Based on developing knowledge of AF mechanisms, various strategies were proposed [10]. Initial studies showed variable efficacy with linear lesions[11], posterior wall isolation[12, 13], left atrial appendage isolation[14–16], targeting CFAEs[17–19] and non-PV triggers[20, 21]. In contrast, other studies[22–24] suggested that most of these strategies did not improve outcomes over PVI alone, a finding eventually confirmed in the STAR AF II trial[3], randomized comparison of PVI, PVI with additional empiric linear, or CFAE ablation, with a trend towards better outcomes with PVI alone.

### Mechanisms and mapping of AF

Focal firing has been recognized as an arrhythmia mechanism for over a century[25, 26], but the initiation of AF through rapid PV activity was described in 1998[27]. Subsequent studies showed that perpetuation of AF may not be totally random, with rapidly changing wavefronts driven by high-frequency localized re-entry demonstrated by high-resolution optical mapping in sheep atria[28].

Focal impulse and rotor modulation (FIRM) technology[29] suggested the presence of rotors and focal impulses in nearly all patients with AF, as did epicardial ECG-based imaging (ECGI)[30]. Targeting such areas of organized activation was associated with termination[29] or organization of AF in remote atrial regions[31]. While the methodology of FIRM was similar to the algorithm used in CARTOFINDER, the main drawback of this technique was relatively poor resolution due to global mapping with a 64-pole basket catheter. This allowed the creation of only a single map of the whole chamber, showing an approximation of a wavefront propagation which was likely not very realistic given the rapid and dynamic change of beat-to-beat wavefront direction in AF.

### Global low resolution vs. sequential high-resolution mapping with CARTOFINDER

#### Global mapping

Previous CARTOFINDER studies using a 64-pole basket catheter showed promising short-term results [6, 32, 33]. Basket catheters can potentially map most of the atrium [34, 35], but global, simultaneous mapping comes at the cost of incomplete electrode contact and low (4–10 mm) resolution. Similar to ECGI, no stable rotors were identified, but transient focal and rotational sources were observed that recurred frequently at the same sites.

#### High-resolution mapping

While recognizing potential limitations with sequential mapping of constantly changing arrhythmias, high-resolution mapping with CARTOFINDER over 30 s at every location allows identification of temporal trends in FAP and RAP. Data acquisition, interpretation and treatment based on this approach do not appear to lengthen procedural duration compared to CC. Potential limitations of sequential recordings with a limited field of view may in the future be mitigated using catheters with additional electrodes and longer splines that enhance coverage without compromising contact or resolution.

#### Repetitive atrial activation patterns

Previous studies using different methodologies indicated a variable prevalence of focal and rotational activation patterns. While our study shows that the automatically identified number of FAP is markedly higher than RAP, in keeping with earlier studies with CARTOFINDER[33, 36, 37], both FIRM[29] and ECGI[30] demonstrated a higher prevalence of rotors than focal breakthroughs: 70% vs. 80% for rotors and 30 vs. 20% for focal firing, respectively, with FIRM[35] suggesting greater spatial stability than ECGI. Predominant locations of the RAAPs identified in this study are similar to those found with ECGI[30], while FIRM did not specify them[29, 35]. The number of RAPs identified in this study was, indeed, lower than with the other methods. Reasons may include several factors including the requirement for head meets tail criteria to be met in the rotational algorithm, the rejection of potential focal sources if arising from the most distal spline of the Pentaray catheter to ensure true focal activity and not activity coming into the “field of view” of the catheter, and the fact that only the left atrium was mapped. There of course has not been a formal direct comparison between specificity and sensitivity of these techniques, and thresholds for stability required to declare an area as a source of RAP vary by technique. There has been wide variability in the frequency of observed RAAPs (both focal and rotational) between different studies and variability in the association between duration of AF and the number of left atrial sources. The total number of reported RAAPs clearly represents the observed number of left *and* right atrial sources as well as both focal and rotational sources, albeit with a majority being rotational and a majority being left atrial with numbers varying between 1.08 and 2.4 per patient[29, 38–40]. Some studies have particularly focused on reporting rotors as the more dominant source.

The intensity of a RAAP source is represented in Cartofinder by the number of repetitions and it varied widely within and between patients. The lower number of repetitions in the rotational sources may result from a very robust algorithm that rejects all sources with incomplete rotation around the pivot point. Moreover, while high-resolution mapping may enhance detection of focal sources and micro re-entry, it also has the potential to miss larger rotational circuits if the Pentaray is not applied at the centre of the circuit. Imminent improvements in catheter technology that will maintain resolution while enhancing “field of view” per application will address this and again in the core of the design of future studies.

#### Comparative efficacy

Cumulative AF-free survival in our CARTOFINDER population was 94% after a single IM procedure at a 1-year follow-up. This appears to be favourable to 1-year results in STAR-AF II (approximately 61%, 54% and 50% in the PVI alone, PVI + CFAE and PVI + linear ablation groups respectively)[3]. Moreover, STAR-AF II had a significant redo rate that varied between 21 and 33% depending on the subgroup.

Although numbers are small, our results seem to be at least comparable to studies involving other individualized mapping technologies. The multicentre FIRM registry showed single‐procedure freedom from AF after a follow‐up of 1 year of approximately 80% in patients with PsAF[41]. However, a subsequent randomized study did not confirm the convincing benefit. STAR global mapping using a basked offers a different approach, focusing on identifying sites that most frequently demonstrate the earliest activation instead of visually depicting wavefront propagation. During a minimum follow-up of 12 months, 80% of patients were free from arrhythmia[42]. Ablation guided by ECGI targeting driver regions was associated with 64% patients remaining in sinus rhythm after 1 year[30], although 59% required AAD and 18% redo procedures. In a later multi-centre study, 77% of patients were free from AF at 1 year, but 49% developed AT requiring further management at follow-up[43], in contrast to 15% in the present study.

#### Study limitations

This study has the following potential limitations: (1) it is a single-centre, nonrandomized, retrospective analysis, although the IM approach was prospectively designed based on an earlier feasibility study; (2) while only two operators performed the procedures, both the visual and automated analysis of data and design of lesion sets are straightforward and widely applicable with minimal additional training for experienced electrophysiologists; (3) follow-up was based on Holter ECG recordings, ECGs and symptomatic review rather than implantable recorders, but this was true of both the IM and CC groups. Thus, although the majority of patients presented with sustained forms of arrhythmia recurrence (AF and AT), some asymptomatic non-sustained arrhythmia episodes may have been missed in both groups. (4) Finally, mapping and ablation were limited to the left atrium.

## Conclusions

Individualized, high-resolution arrhythmia mapping to guide ablation beyond PVI based on CARTOFINDER shows much promise if applied as a primary treatment for the first-time ablation. There is a pressing need to improve outcomes in patients undergoing ablation for PsAF and LsPsAF, and the promising results of this pilot study need to be confirmed in a larger, randomized trial.

## Supplementary Information

Below is the link to the electronic supplementary material.

Supplementary file1 (MPG 59314 KB)

Supplementary file2 (MPG 50144 KB)

## Data Availability

The data underlying this article will be shared at reasonable request to the corresponding author.

## References

[1] Pappone C, Vicedomini G, Augello G, Manguso F, Saviano M, Baldi M (2011). Radiofrequency catheter ablation and antiarrhythmic drug therapy: A prospective, randomized, 4-year follow-up trial: The APAF study. Circ Arrhythmia Electrophysiol.

[2] Rostock T, Salukhe TV, Steven D, Drewitz I, Hoffmann BA, Bock K (2011). Long-term single- and multiple-procedure outcome and predictors of success after catheter ablation for persistent atrial fibrillation. Heart Rhythm.

[3] Verma A, Jiang CY, Betts TR, Chen J, Deisenhofer I, Mantovan R (2015). Approaches to catheter ablation for persistent atrial fibrillation. N Engl J Med.

[4] Boyalla V, Jarman JWE, Markides V, Hussain W, Wong T, Mead RH, et al. Internationally validated score to predict the outcome of non-paroxysmal atrial fibrillation ablation: The “FLAME score.” Open Heart. 2021;8. 10.1136/openhrt-2021-001653 PMC8340273 34348972

[5] Haïssaguerre M, Sanders P, Hocini M, Hsu LF, Shah DC, Scavée C (2004). Changes in atrial fibrillation cycle length and inducibility during catheter ablation and their relation to outcome. Circulation.

[6] Honarbakhsh S, Schilling RJ, Dhillon G, Ullah W, Keating E, Providencia R (2018). A Novel Mapping System for Panoramic Mapping of the Left Atrium: Application to Detect and Characterize Localized Sources Maintaining Atrial Fibrillation. JACC Clin Electrophysiol.

[7] Honarbakhsh S, Hunter RJ, Dhillon G, Ullah W, Keating E, Providencia R (2018). Validation of a novel mapping system and utility for mapping complex atrial tachycardias. J Cardiovasc Electrophysiol.

[8] Daoud EG, Zeidan Z, Hummel JD, Weiss R, Houmsse M, Augostini R (2017). Identification of Repetitive Activation Patterns Using Novel Computational Analysis of Multielectrode Recordings During Atrial Fibrillation and Flutter in Humans. JACC Clin Electrophysiol.

[9] Winkle RA, Moskovitz R, Hardwin Mead R, Engel G, Kong MH, Fleming W (2018). Atrial fibrillation ablation using very short duration 50 W ablations and contact force sensing catheters. J Interv Card Electrophysiol.

[10] Zaman JAB, Schricker A, Lalani GG, Trikha R, Krummen DE, Narayan SM. Focal impulse and rotor mapping (firm): Conceptualizing and treating atrial fibrillation. J. Atr. Fibrillation. 2014. p. 1103. 10.4022/jafib.1103 PMC5135257 27957100

[11] Gaita F, Caponi D, Scaglione M, Montefusco A, Corleto A, Di Monte F (2008). Long-term clinical results of 2 different ablation strategies in patients with paroxysmal and persistent atrial fibrillation. Circ Arrhythm Electrophysiol.

[12] Saad EB, Slater C (2014). Complete isolation of the left atrial posterior wall (Box Lesion) to treat longstanding persistent atrial fibrillation. J Atr Fibrillation.

[13] Reddy VY, Neuzil P, D’Avila A, Ruskin JN (2008). Isolating the posterior left atrium and pulmonary veins with a “box” lesion set: Use of epicardial ablation to complete electrical isolation. J Cardiovasc Electrophysiol.

[14] Panikker S, Jarman JWE, Virmani R, Kutys R, Haldar S, Lim E (2016). Left atrial appendage electrical isolation and concomitant device occlusion to treat persistent atrial fibrillation. Circ Arrhythmia Electrophysiol..

[15] Di Biase L, Burkhardt JD, Mohanty P, Mohanty S, Sanchez JE, Trivedi C (2016). Left Atrial Appendage Isolation in Patients With Longstanding Persistent AF Undergoing Catheter Ablation: BELIEF Trial. J Am Coll Cardiol.

[16] Di Biase L, Burkhardt JD, Mohanty P, Sanchez J, Mohanty S, Horton R (2010). Left atrial appendage: An underrecognized trigger site of atrial fibrillation. Circulation.

[17] Schmitt C, Estner H, Hecher B, Luik A, Kolb C, Karch M (2007). Radiofrequency ablation of complex fractionated atrial electrograms (CFAE): Preferential sites of acute termination and regularization in paroxysmal and persistent atrial fibrillation. J Cardiovasc Electrophysiol.

[18] Porter M, Spear W, Akar JG, Helms R, Brysiewicz N, Santucci P (2008). Prospective study of atrial fibrillation termination during ablation guided by automated detection of fractionated electrograms. J Cardiovasc Electrophysiol.

[19] Nademanee K, Lockwood E, Oketani N, Gidney B (2010). Catheter ablation of atrial fibrillation guided by complex fractionated atrial electrogram mapping of atrial fibrillation substrate. J Cardiol.

[20] Corrado A, Bonso A, Madalosso M, Rossillo A, Themistoclakis S, Di Biase L (2010). Impact of systematic isolation of superior vena cava in addition to pulmonary vein antrum isolation on the outcome of paroxysmal, persistent, and permanent atrial fibrillation ablation: Results from a randomized study. J Cardiovasc Electrophysiol.

[21] Zhao Y, Di Biase L, Trivedi C, Mohanty S, Bai R, Mohanty P (2016). Importance of non-pulmonary vein triggers ablation to achieve long-term freedom from paroxysmal atrial fibrillation in patients with low ejection fraction. Heart Rhythm.

[22] Tamborero D, Mont L, Berruezo A, Matiello M, Benito B, Sitges M (2009). Left atrial posterior wall isolation does not improve the outcome of circumferential pulmonary vein ablation for atrial fibrillation: A prospective randomized study. Circ Arrhythmia Electrophysiol.

[23] Oral H, Chugh A, Good E, Wimmer A, Dey S, Gadeela N (2007). Radiofrequency catheter ablation of chronic atrial fibrillation guided by complex electrograms. Circulation.

[24] Wynn GJ, Panikker S, Morgan M, Hall M, Waktare J, Markides V (2016). Biatrial linear ablation in sustained nonpermanent AF: Results of the substrate modification with ablation and antiarrhythmic drugs in nonpermanent atrial fibrillation (SMAN-PAF) trial. Heart Rhythm.

[25] Winterberg H. Studien über Herzflimmern - I. Mitteilung. Über die Wirkung des N. vagus und accelerans auf das Flimmern des Herzens. Pflüger, Arch für die Gesammte Physiol des Menschen und der Thiere. 1907;117:223–56.

[26] Lee S, Khrestian CM, Sahadevan J, Waldo AL (2020). Reconsidering the multiple wavelet hypothesis of atrial fibrillation. Heart Rhythm.

[27] Haïssaguerre M, Jaïs P, Shah DC, Takahashi A, Hocini M, Quiniou G (1998). Spontaneous initiation of atrial fibrillation by ectopic beats originating in the pulmonary veins. N Engl J Med.

[28] Mansour M, Mandapati R, Berenfeld O, Chen J, Samie FH, Jalife J (2001). Left-to-right gradient of atrial frequencies during acute atrial fibrillation in the isolated sheep Heartt. Circulation.

[29] Narayan SM, Krummen DE, Shivkumar K, Clopton P, Rappel WJ, Miller JM (2012). Treatment of atrial fibrillation by the ablation of localized sources: CONFIRM (Conventional Ablation for Atrial Fibrillation with or Without Focal Impulse and Rotor Modulation) trial. J Am Coll Cardiol.

[30] Haissaguerre M, Hocini M, Denis A, Shah AJ, Komatsu Y, Yamashita S (2014). Driver domains in persistent atrial fibrillation. Circulation.

[31] Jarman JWE, Wong T, Kojodjojo P, Spohr H, Davies JER, Roughton M (2014). Organizational index mapping to identify focal sources during persistent atrial fibrillation. J Cardiovasc Electrophysiol.

[32] Honarbakhsh S, Schilling RJ, Providencia R, Keating E, Chow A, Sporton S (2018). Characterization of drivers maintaining atrial fibrillation: Correlation with markers of rapidity and organization on spectral analysis. Heart Rhythm.

[33] Verma A, Sarkozy A, Skanes A, Duytschaever M, Bulava A, Urman R (2018). Characterization and significance of localized sources identified by a novel automated algorithm during mapping of human persistent atrial fibrillation. J Cardiovasc Electrophysiol.

[34] Rensma PL, Allessie MA, Lammers WJEP, Bonke FIM, Schalij MJ (1988). Length of excitation wave and susceptibility to reentrant atrial arrhythmias in normal conscious dogs. Circ Res.

[35] Narayan SM, Krummen DE, Rappel WJ (2012). Clinical mapping approach to diagnose electrical rotors and focal impulse sources for human atrial fibrillation. J Cardiovasc Electrophysiol.

[36] Wolf M, Tavernier R, Zeidan Z, El Haddad M, Vandekerckhove Y, De PJ (2019). Identification of repetitive atrial activation patterns in persistent atrial fibrillation by direct contact high-density electrogram mapping. J Cardiovasc Electrophysiol.

[37] Takahashi Y, Akiyoshi K, Sekigawa M, Yagishita A, Yamamoto T, Maeda S (2020). Endocardial contact mapping of the left atrial appendage in persistent atrial fibrillation. J Cardiovasc Electrophysiol.

[38] Buch E, Share M, Tung R, Benharash P, Sharma P, Koneru J (2016). Long-term clinical outcomes of focal impulse and rotor modulation for treatment of atrial fibrillation: A multicenter experience. Heart Rhythm.

[39] Swarup V, Baykaner T, Rostamian A, Daubert JP, Hummel J, Krummen DE (2014). Stability of rotors and focal sources for human atrial fibrillation: Focal impulse and rotor mapping (firm) of AF sources and fibrillatory conduction. J Cardiovasc Electrophysiol.

[40] Kirzner JM, Raelson CA, Liu CF, Thomas G, Ip JE, Lerman BB (2019). Effects of focal impulse and rotor modulation-guided ablation on atrial arrhythmia termination and inducibility: Impact on outcomes after treatment of persistent atrial fibrillation. J Cardiovasc Electrophysiol.

[41] Miller JM, Kowal RC, Swarup V, Daubert JP, Daoud EG, Day JD (2014). Initial independent outcomes from focal impulse and rotor modulation ablation for atrial fibrillation: Multicenter FIRM registry. J Cardiovasc Electrophysiol.

[42] Honarbakhsh S, Hunter RJ, Ullah W, Keating E, Finlay M, Schilling RJ (2019). Ablation in Persistent Atrial Fibrillation Using Stochastic Trajectory Analysis of Ranked Signals (STAR) Mapping Method. JACC Clin Electrophysiol.

[43] Knecht S, Sohal M, Deisenhofer I, Albenque JP, Arentz T, Neumann T (2017). Multicentre evaluation of non-invasive biatrial mapping for persistent atrial fibrillation ablation: The AFACART study. Europace.

